# Combinatorial Pattern of Histone Modifications in Exon Skipping Event

**DOI:** 10.3389/fgene.2019.00122

**Published:** 2019-02-18

**Authors:** Wei Chen, Xiaoming Song, Hao Lin

**Affiliations:** ^1^Innovative Institute of Chinese Medicine and Pharmacy, Chengdu University of Traditional Chinese Medicine, Chengdu, China; ^2^Center for Genomics and Computational Biology, School of Life Sciences, North China University of Science and Technology, Tangshan, China; ^3^Key Laboratory for Neuro-Information of Ministry of Education, Center for Informational Biology, School of Life Sciences and Technology, University of Electronic Science and Technology of China, Chengdu, China

**Keywords:** histone modification, methylation, acetylation, RNA splicing, Bayesian network, casual relationship

## Abstract

Histone modifications are associated with alternative splicing. It has been suggested that histone modifications act in combinational patterns in gene expression regulation. However, how they interact with each other and what is their casual relationships in the process of RNA splicing remain unclear. In this study, the combinatorial patterns of 38 kinds of histone modifications in the exon skipping event of the CD4^+^ T cell were analyzed by constructing Bayesian networks. Distinct combinatorial patterns of histone modifications that illustrating their casual relationships were observed in excluded/included exons and the surrounding intronic regions. The Bayesian networks also indicate that some histone modifications directly correlate with RNA splicing. We anticipate that this work could provide novel insights into the effects of histone modifications on RNA splicing regulation.

## Introduction

Alternative splicing is a process that can generate multiple mRNA isoforms from a single gene by splicing pre-mRNA molecules in different ways (Black, 2003). As an important process of gene expression, alternative splicing ensures the diversity of gene expression products. It has been estimated that alternative splicing occurs in approximately 90% human genes (Pan et al., 2008; Wang E. T. et al., 2008). Alternative splicing is reported to closely correlate with apoptosis, embryonic development and even a series of diseases (Garcia-Blanco et al., 2004; David et al., 2010; Lai and Greenberg, 2013; Scotti and Swanson, 2016). Although great efforts have been made on studying alternative splicing, the mechanisms of cell type-specific and stage-specific alternative splicing are still unclear (Nellore et al., 2016).

Recent studies have revealed that alternative splicing is regulated not only by *trans*-acting factors that can interact with cis-acting elements (Badr and Heath, 2015; Badr et al., 2016), but also by epigenetic factors, such as DNA methylation, nucleosome occupancy, and so on (Mele et al., 2017). Since RNA splicing is coupled to transcription, histone modifications were also found to be involved in alternative splicing regulation (Kornblihtt et al., 2013). Luco et al. (2010) found that the alternative splicing of the FGFR2 gene was correlated with the level of H3K36me3. Saint-Andre et al. (2011) demonstrated that the inclusion/exclusion of the alternative exons of the CD44 mRNA is affected by H3K9me3. The combinatorial effect of histone modifications on alternative splicing was also reported. Recently, Shindo et al. (2013) found that the alternative splicing of the BIN1 gene in IMR90 cell was regulated by the cooperation of H3K36me3, H3K4me3, H2BK12ac, and H4K5ac. These results strongly indicate that histone modifications play important roles in RNA splicing regulation and are key clues for revealing the regulatory mechanism of alternative splicing.

Based on these experimental results, several computational methods have been proposed to predict the alternative exons in exon skipping event based on histone modifications. The pioneer work was proposed by Enroth et al. (2012), in which a rule-based model was developed to classify included and excluded exons based on histone modification combinations. Later on, based on Enroth et al.’s (2012) dataset, Chen et al. (2014) proposed a quadratic discriminant (QD) function method and obtained an accuracy of 68.5% for classifying the included and excluded exons in the exon skipping event. More recently, a random forest based method was developed for the same aim and obtained an accuracy of 72.91% in the 10-fold cross validation test (Chen et al., 2018b). These results strongly indicate that histone modifications play important roles in RNA splicing regulation and are key clues for revealing the regulatory mechanism of alternative splicing. These results indicate that we should find the novel splicing code from the epigenome information.

Inspired by recent works (Cui et al., 2011; Zhu et al., 2013), in this study, the Bayesian network of histone modifications were constructed in the excluded/included exons and their preceding and succeeding intronic regions of exon skipping event to investigate how histone modifications interact with each other and find their casual relationships in the process of RNA splicing. By analyzing the Bayesian networks, distinct combinational patterns and casual relationships of histone modifications were observed in different regions relative to exons.

## Materials and Methods

### Dataset

Based on the exon expression data of the CD4^+^ T cell (Oberdoerffer et al., 2008), by calculating the ratio between exon expression and gene expression, Enroth et al. (2012) obtained 13,374 “included” and 11,587 “excluded” exons. All of these exons are longer than 50 bp with flanking introns longer than 360 bp, and none of them are the first or last exon in any transcripts (Enroth et al., 2012).

The ChIP-seq data for the 20 kinds of histone methylations (H3K27me2, H3K4me1, H3K79me2, H3K9me3, H4K20me3, H3K27me3, H3K4me2, H3K79me3, H3R2me1, H4R3me2, H2BK5me1, H3K36me1, H3K4me3, H3K9me1, H3R2me2, H3K27me1, H3K36me3, H3K79me1, H3K9me2, and H4K20me1) and 18 kinds of histone acetylation modifications (H2AK5ac, H2BK20ac, H3K23ac, H3K9ac, H4K8ac, H2AK9ac, H2BK5ac, H3K27ac, H4K12ac, H4K91ac, H2BK120ac, H3K14ac, H3K36ac, H4K16ac, H2BK12ac, H3K18ac, H3K4ac, and H4K5ac) of the CD4^+^ T cell were obtained from previous works (Barski et al., 2007; Wang Z. et al., 2008). By using the SICTIN tool, Enroth et al. (2010) discretized the histone modification signals to binary (present/absent) attributes over the three regions, namely excluded/included exons, the closest 180 bp flanking intronic regions proceeding and succeeding the exons.

After winnowing out exons with no modifications present, they finally obtained 12,692 “included” and 11,165 “excluded” exons. The present/absent of the 38 kinds of histone modifications in the excluded/included exon and the preceding and succeeding intronic regions was annotated by “1” (indicating the presence of histone modification) or “0” (indicating the absence of histone modification), which were used to construct the histone modification Bayesian network. All the data can be found in Enroth et al.’s (2010) work.

### Bayesian Network

Bayesian network is a probabilistic graphical model that represents a set of variables and their conditional dependencies via a directed acyclic graph (DAG) (Yu et al., 2008). The nodes in Bayesian network represent variables, and the edges represent conditional dependencies. A directed edge (→) from node *a_i_* to node *a_j_* represents a statistical dependence or the causal relationships between the corresponding variables. The arrow indicates that the variable *a_j_* depends on the variable *a_i_*. If there is no edge between two nodes *a_i_* and *a_j_*, indicating that the variables *i* and *j* are independent of each other.

In this study, the WinMine package which is available at https://www.microsoft.com/en-us/research/project/winmine-toolkit/#!downloads, was used to construct the Bayesian network of histone modifications in the excluded/included exons and the preceding and succeeding intronic regions. The nodes in the potential networks will be the histone modifications.

## Results and Discussion

### Correlations Between Histone Modifications

Previous studies have reported that gene expression is in part regulated by histone modifications that act in a combinatorial fashion, i.e., the so-called “histone code” (Yu et al., 2008; Cui et al., 2011; Zhu et al., 2013). In order to find whether the combinatorial pattern of histone modifications exist in the process of RNA splicing, we first calculated the Pearson correlation coefficients between the 38 kinds of histone modifications in the excluded/included exons and the preceding and succeeding intronic regions, respectively.

**FIGURE 1:**
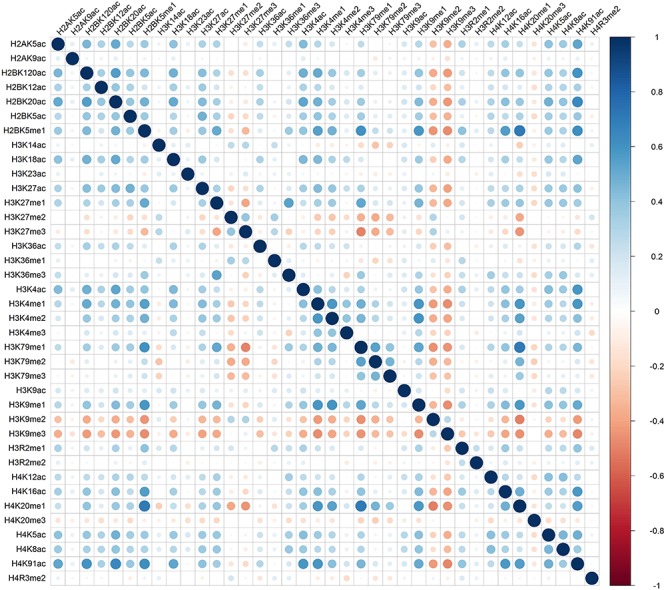
The heatmap of Pearson correlation coefficients of histone modifications in the included exon.

**FIGURE 2:**
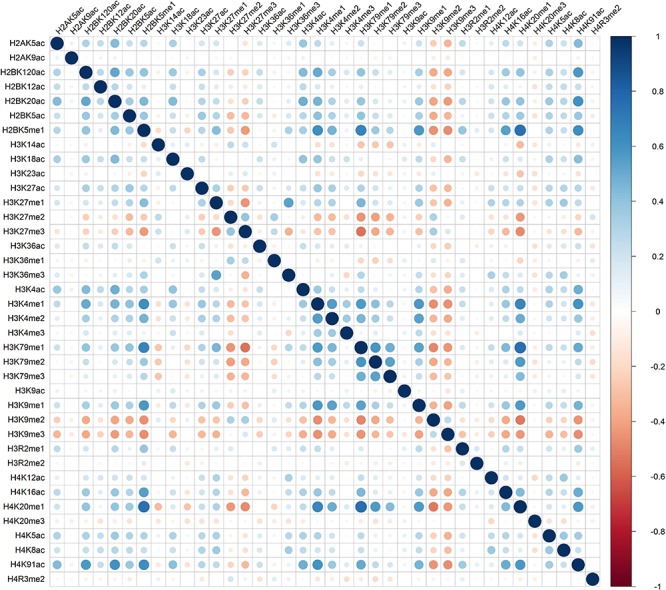
The heatmap of Pearson correlation coefficients of histone modifications in the excluded exon.

Distinct combinatorial patterns of histone modifications were observed in the excluded/included exon and the surrounding regions. For example, H2BK5me1 was found to be positively correlated with H3K4me1, H3K4me2, H3K79me1, H3K9me1, H4K20me1, and H4K91ac in both included and excluded exons, Figure 1 and 2. The negative relationship were found between H3K9me3 with most of the remaining 37 kinds of histone modifications in both excluded and included exons, Figure 1 and 2. These results also hold for the preceding and succeeding intronic regions of the included and excluded exons (Supplementary Figures S1–S4).

Besides the common pattern, the excluded/included exon specific combinatorial patterns of histone modifications were also observed in excluded/included exon, Figure 1 and 2. For example, in the included exon, H3K4ac and H2BK5me1, H3K79me1 and H3K23ac, H4K20ac and H3K4me1, H4K20ac and H3K4me2 exhibit negative correlations, which is absent in excluded exon; while the significantly negative correlation between H3K14ac and H2BK5me1, H4K120ac and H3K27me2, H4K120ac and H3K27me2 were only observed in the excluded exon. The excluded/included exon specific combinatorial patterns of histone modifications can also be found in the preceding and succeeding intronic regions of the included and excluded exons (Supplementary Figures S1–S4).

### Interaction Network of Histone Modifications

In order to investigate how histone modifications interact with each other and how their combinational fashions regulate RNA splicing, the Bayesian networks were constructed to deduce the causal relationships among histone modifications in the excluded/included exons and the surrounding intronic regions, respectively. In the Bayesian network, the nodes are the histone modifications, and the edge from one node to another one is their Pearson correlation coefficient.

The 10-fold cross-validation test method was used to find the robust Bayesian networks (Yu et al., 2008). The detailed procedure is as following. In the 10-fold cross-validation test, the dataset (Materials and Methods) is randomly partitioned into ten subsets, and nine of them were used to generate a Bayesian network. Based on the Pearson correlation coefficient of histone modifications of the nine subsets, a fundamental Bayesian network demonstrating the casual relationship between histone modifications was built by using the WinMine package. The 10-fold cross-validation was repeated 10 times. Accordingly, 10 fundamental Bayesian networks will be obtained for the excluded/included exons and the surrounding intronic regions, respectively. According to previous work (Yu et al., 2008), the final Bayesian network was then constructed based on the 10 fundamental Bayesian networks, in which each edge should be appeared within seven of the 10 fundamental Bayesian networks. The edges in the networks were colored according to the Pearson correlations between the two nodes linked by the edge.

**FIGURE 3:**
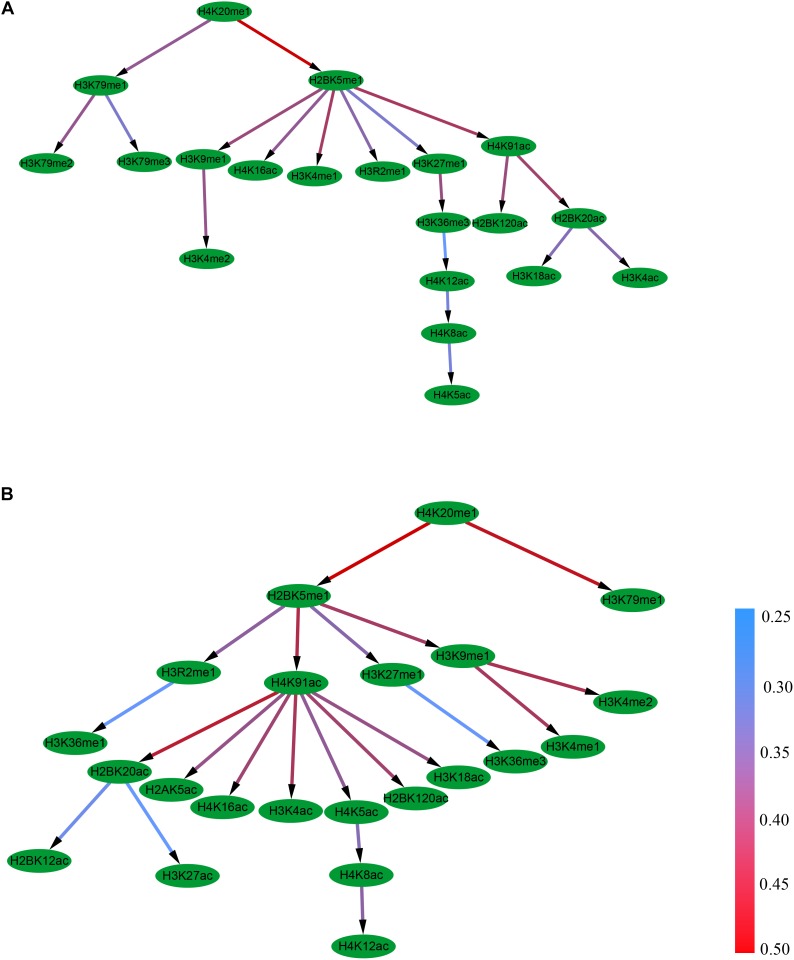
The Bayesian network of histone modifications in the excluded exon **(A)** and included exon **(B)**.

It was found that the Bayesian network for the excluded exon event contains 19 edges and 10 combinational patterns of histone modifications, including interactions between different levels of the same modification (e.g., H3K79me1, H3K79me2, and H3K79me3), between modifications on different amino acids (e.g., H2BK5me1 and H3K9me1), and between different kinds of modifications (e.g., H2BK5me1 and H4K16ac), Figure 3A. It can also be observed that the 10 histone modifications that have direct correlations with RNA splicing in excluded exon are H3K79me3, H3K79me2, H3K4me2, H4K16ac, H3K4me1, H3R2me1, H4K5ac, H2BK120ac, H3K18ac, and H3K4ac.

Distinct from the excluded exon, the Bayesian network for the included exon event contains 21 edges and 13 combinational patterns of histone modifications (Figure 3B). The interactions between modifications on different amino acids (e.g., H4K20me1 and H3K79me1), and between different kinds of modifications (e.g., H2BK5me1 and H4K91ac) were observed in this case. There are 13 histone modifications (H3K79me1, H3K36me3, H3K36me1, H3K4me1, H3K4me2, H2BK12ac, H3K27ac, H2AK5ac, H4K16ac, H3K4ac, H4K12ac, H2BK120ac, and H3K18ac) that have direct correlations with RNA splicing.

The above results demonstrate that the topologies of the Bayesian networks of histone modifications for the included and excluded exon in the skipping event are different. Moreover, the differences also exist in the proceeding and succeeding intronic regions of the included and excluded exons (Supplementary Figures S5–S6). Therefore, it can be concluded that the casual relationship of histone modifications were obviously different between included and excluded exons.

## Conclusion

Based on the Pearson correlation coefficients, the casual relationships of histone modifications in the process of RNA splicing were deduced by constructing their Bayesian networks. The results indicate that the inclusion or exclusion of exons is influenced by combinatorial patterns of histone modifications (Figure 3 and Supplementary Figures S5–S6). Some of the histone modifications contribute directly to RNA splicing (e.g., H3K36me3 and H3K79me1), while other histone modifications indirectly contribute to the RNA splicing.

The result that H3K36me3 and H3K79me1 can affect RNA splicing is consistent with previous studies which have demonstrated that H3K36me3 and H3K79me1 are enriched in included exons (Shindo et al., 2013). The H3K36me3 can regulate alternative splicing by interacting with polypyrimidine tract-binding protein (PTB) (Luco et al., 2010). By interacting with the Tudor domain of TP53BP1, the H3K79me1 was also reported to interact with that interacts with snRNP (Huyen et al., 2004; Shindo et al., 2013).

By relaxing the chromatin structure, the H3 and H4 acetylation were also reported to regulating inclusion or exclusion of the skipping exon (Zhou et al., 2011). Besides the histone modifications located in exon regions, histone modifications located in intragenic regions can also influence RNA splicing by regulating RNAPII elongation rates, or by directly binding to splicing factors and hence mediating their binding to pre-mRNA (Gomez Acuna et al., 2013).

Since there is no evidence for some of the histone modifications how they regulate RNA splicing, further experiments are needed in order to illustrate their roles in RNA splicing regulation. Taken together, we hope that this work could provide novel insights into the research on RNA splicing. Besides histone modifications, the method proposed here could also be used to analyze the relationship between RNA splicing with other modifications, such as m6A (Chen et al., 2015; Chen et al., 2018a; Wei et al., 2019), m4C (Chen et al., 2017; He et al., 2018), phosphorylation (Wei et al., 2017), GlcNAcylation (Jia et al., 2018), etc.

## Author Contributions

WC and HL conceived and designed the experiments. HL, XS, and WC wrote the manuscript. All authors performed the experiments, read, and approved the final manuscript.

## Conflict of Interest Statement

The authors declare that the research was conducted in the absence of any commercial or financial relationships that could be construed as a potential conflict of interest.
