# Supplementary material for: Combinatorial Pattern of Histone Modifications in Exon Skipping Event
Source: Front Genet. 2019 Feb 18;10:122. doi: 10.3389/fgene.2019.00122 (PMC6387913; doi:10.3389/fgene.2019.00122)
Supplement: Supplementary file 1 [file Table_1.DOCX]

**Supplementary Figures**

**Combinatorial pattern of histone modifications in exon skipping event**

**Wei Chen^1, ,3^*, Xiaoming Song^2^, Hao Lin^3^***

1. Innovative Institute of Chinese Medicine and Pharmacy, Chengdu University of Traditional Chinese Medicine, Chengdu 611730, China;
2. Center for Genomics and Computational Biology, School of Life Sciences, North China University of Science and Technology, Tangshan 063000, China;
3. Key Laboratory for Neuro-Information of Ministry of Education, School of Life Science and Technology, Center for Informational Biology, University of Electronic Science and Technology of China, Chengdu, 610054, China

*Corresponding authors

Wei Chen: Tel: +86 315 3725715; Fax: +86 315 3725715; E-mail: [chenweiimu@gmail.com](mailto:chenweiimu@gmail.com);

Hao Lin: Tel: +86 28 8320 2351; Fax: +86 28 8320 8238; E-mail: [hlin@uestc.edu.cn](mailto:hlin@uestc.edu.cn).

**Running Title:** Combinatorial pattern of histone modifications


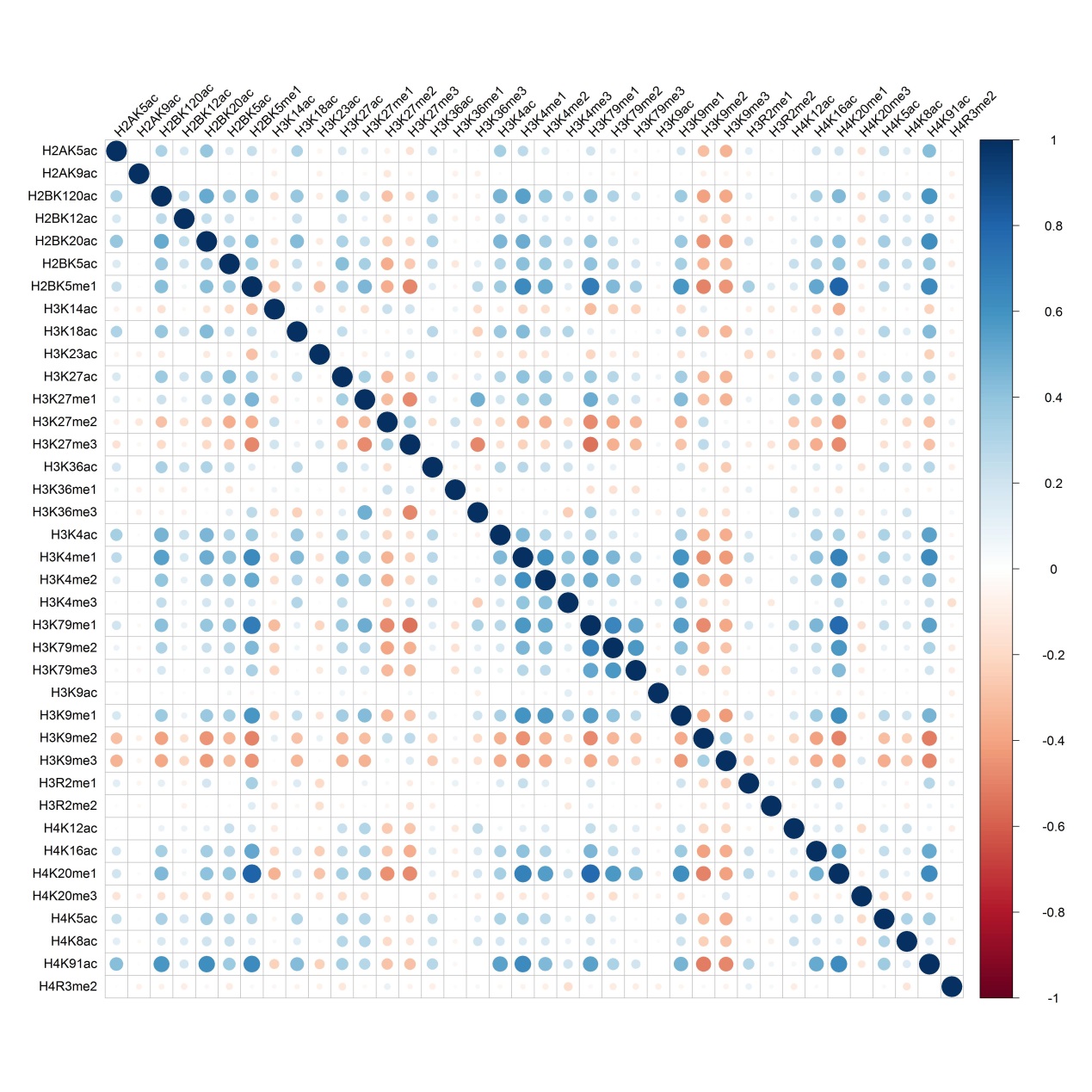


**Supplementary Figure S1** The heatmap of Pearson correlation coefficients of histone modifications in the proceeding intronic regions of the excluded exon.


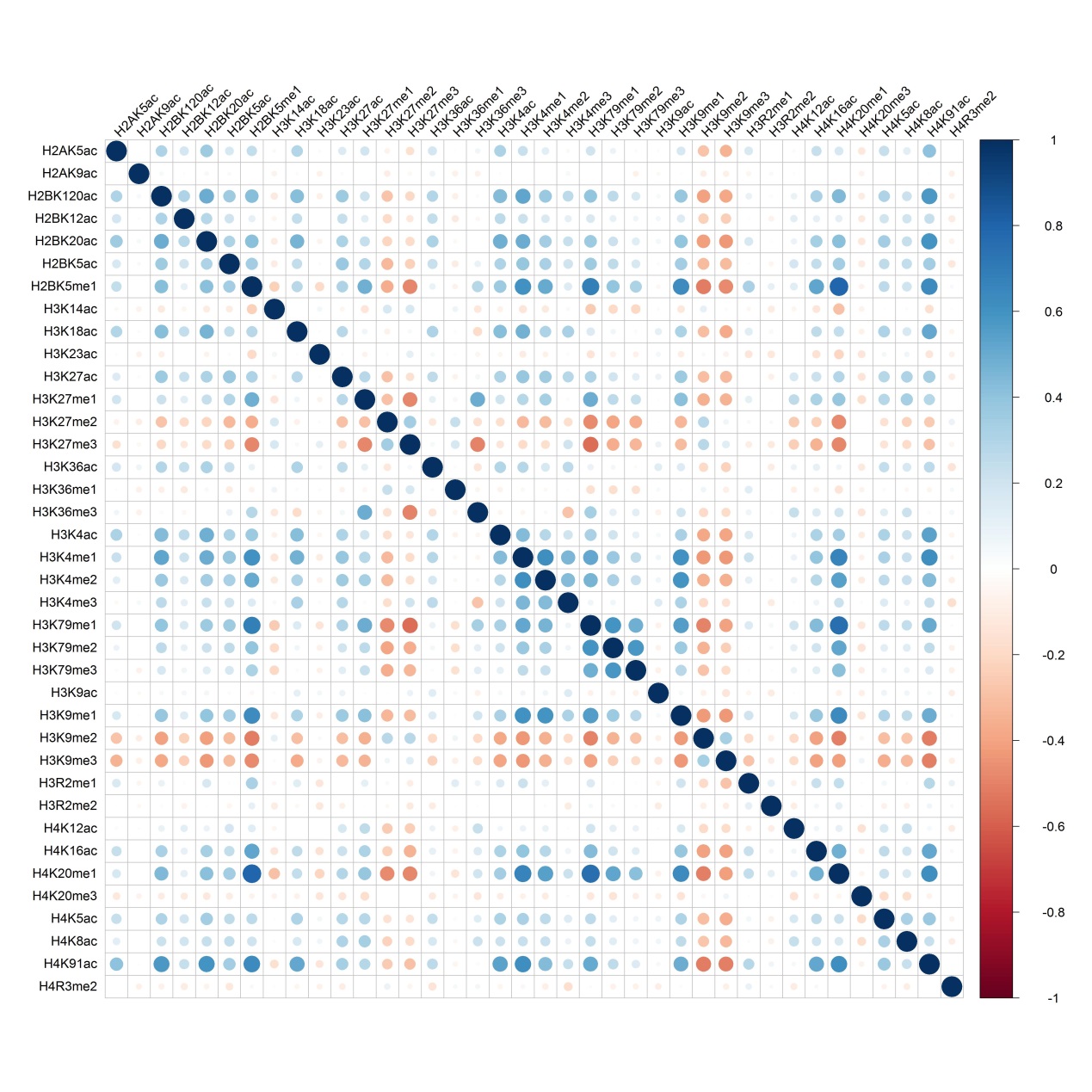


**Supplementary Figure S2** The heatmap of Pearson correlation coefficients of histone modifications in the proceeding intronic regions of the included exon.


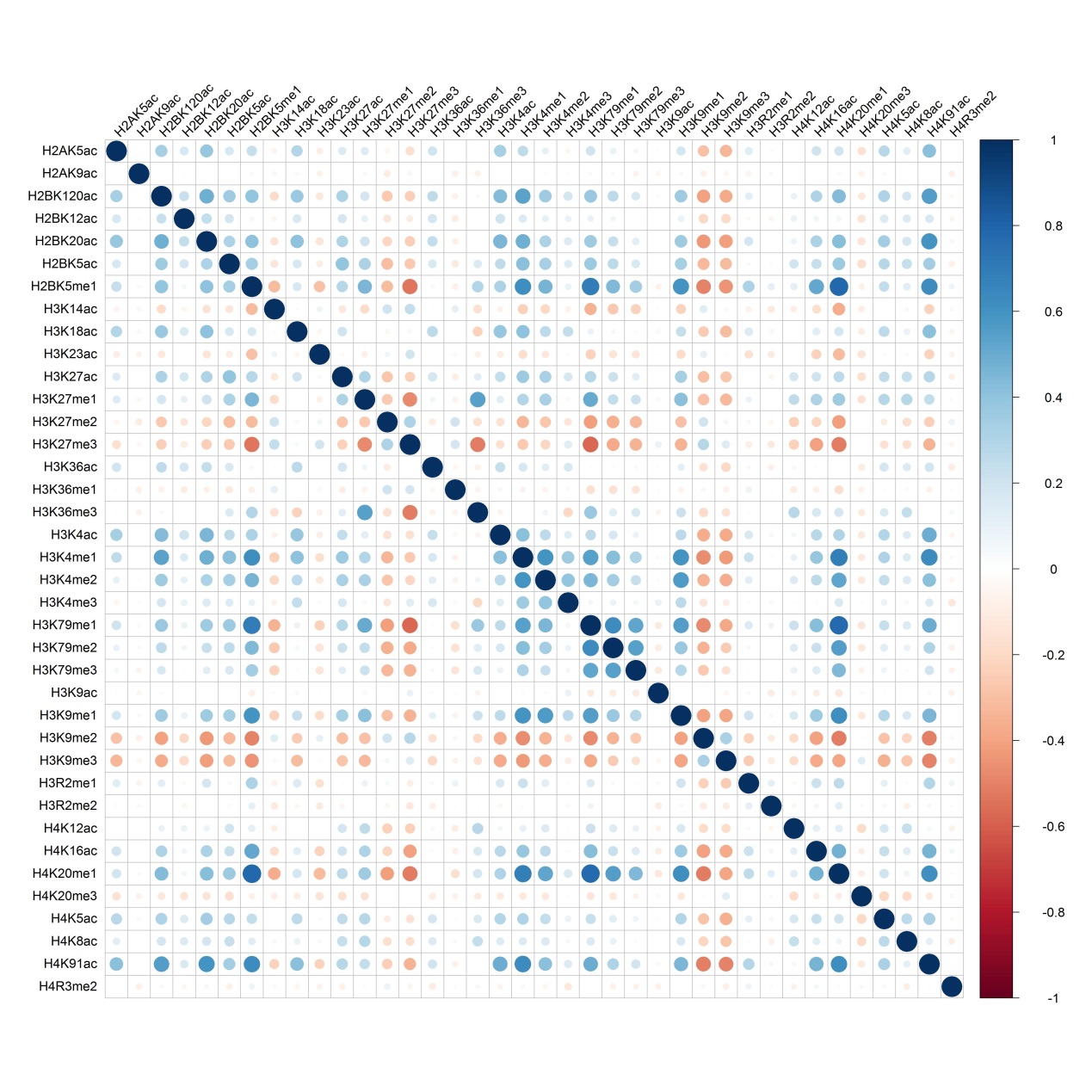


**Supplementary Figure S3** The heatmap of Pearson correlation coefficients of histone modifications in the succeeding intronic regions of the excluded exon.


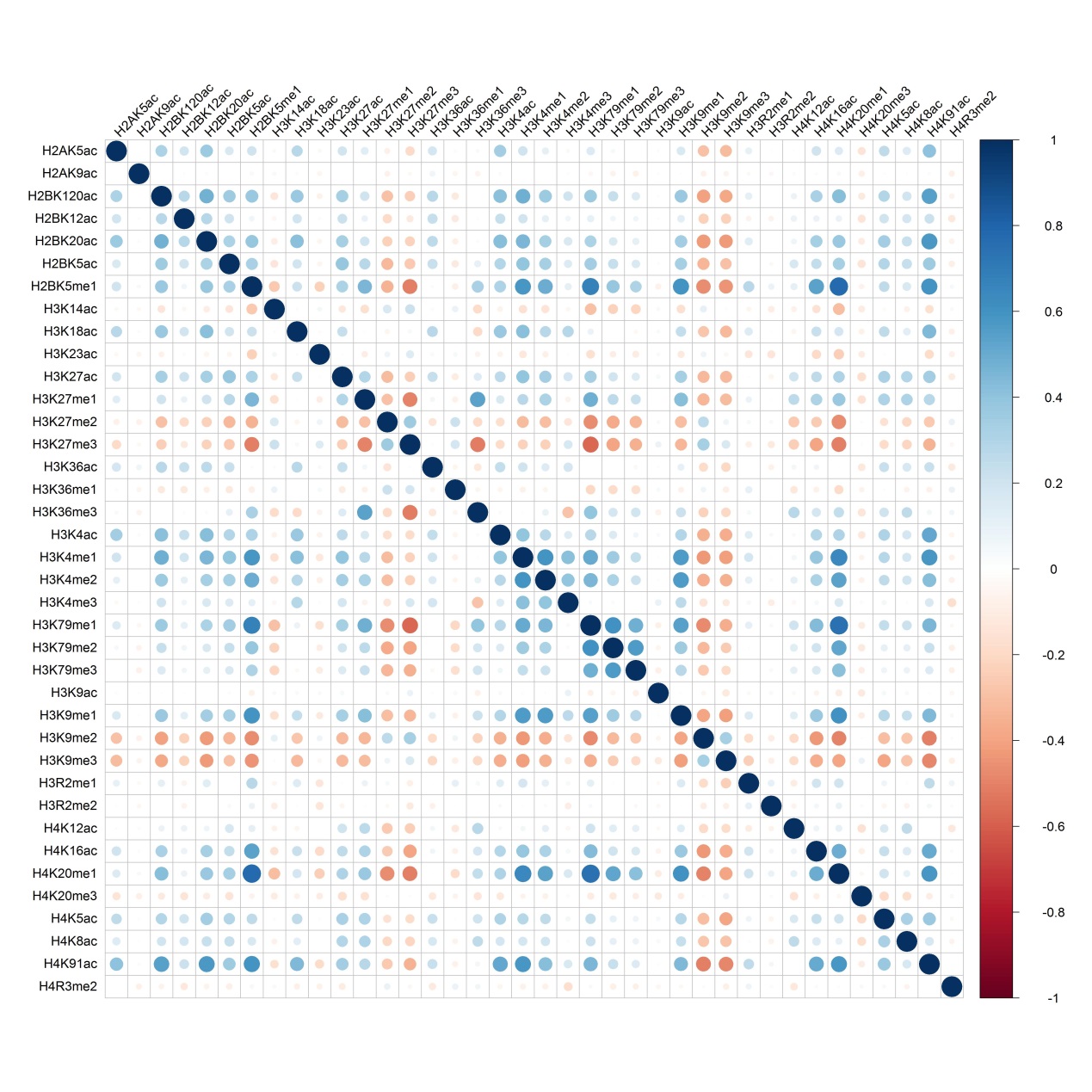


**Supplementary Figure S4** The heatmap of Pearson correlation coefficients of histone modifications in the succeeding intronic regions of the included exon.


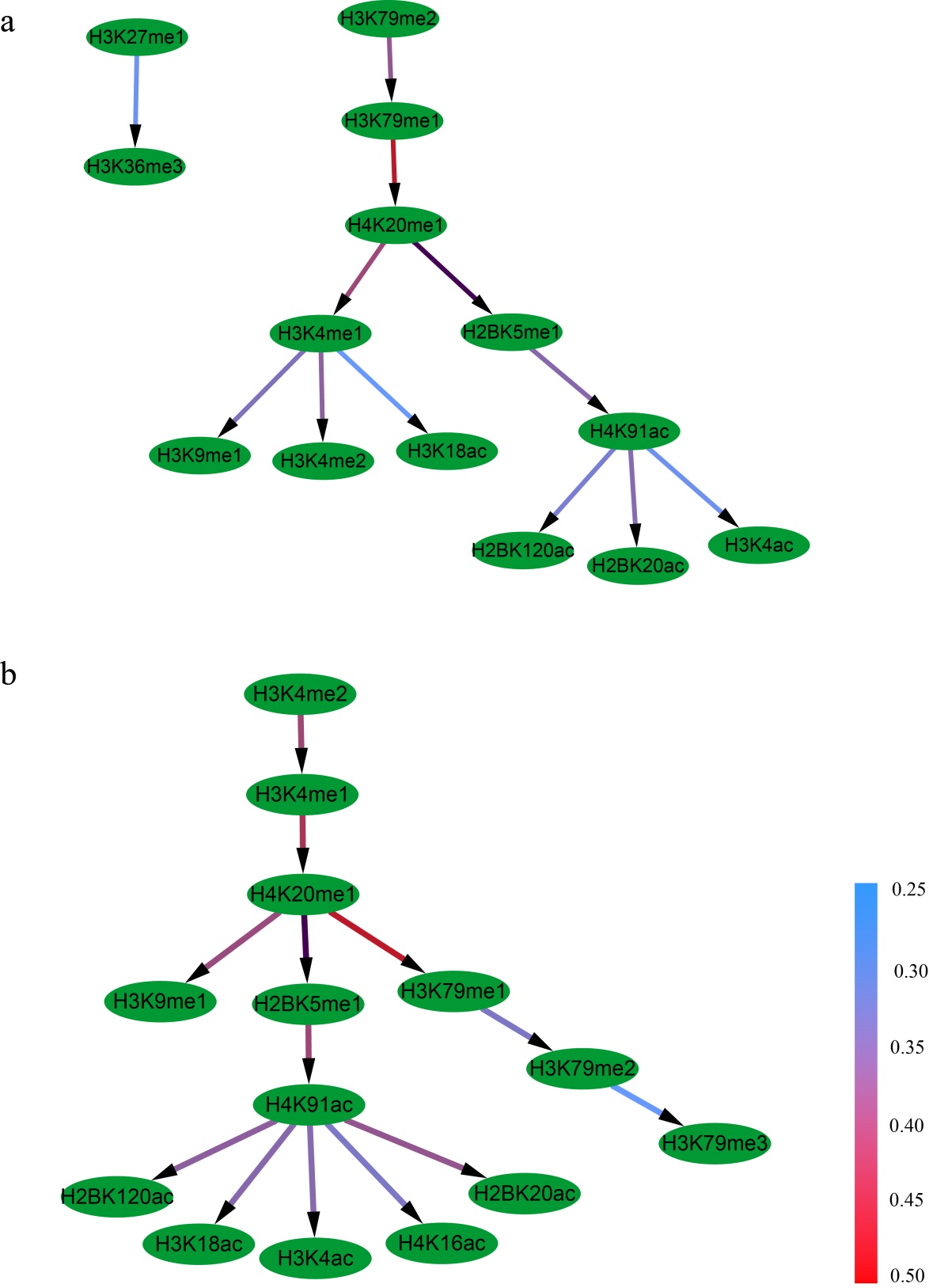


**Figure S5** The Bayesian network of histone modifications in proceeding intronic region of the excluded exon (a) and excluded exon (b).


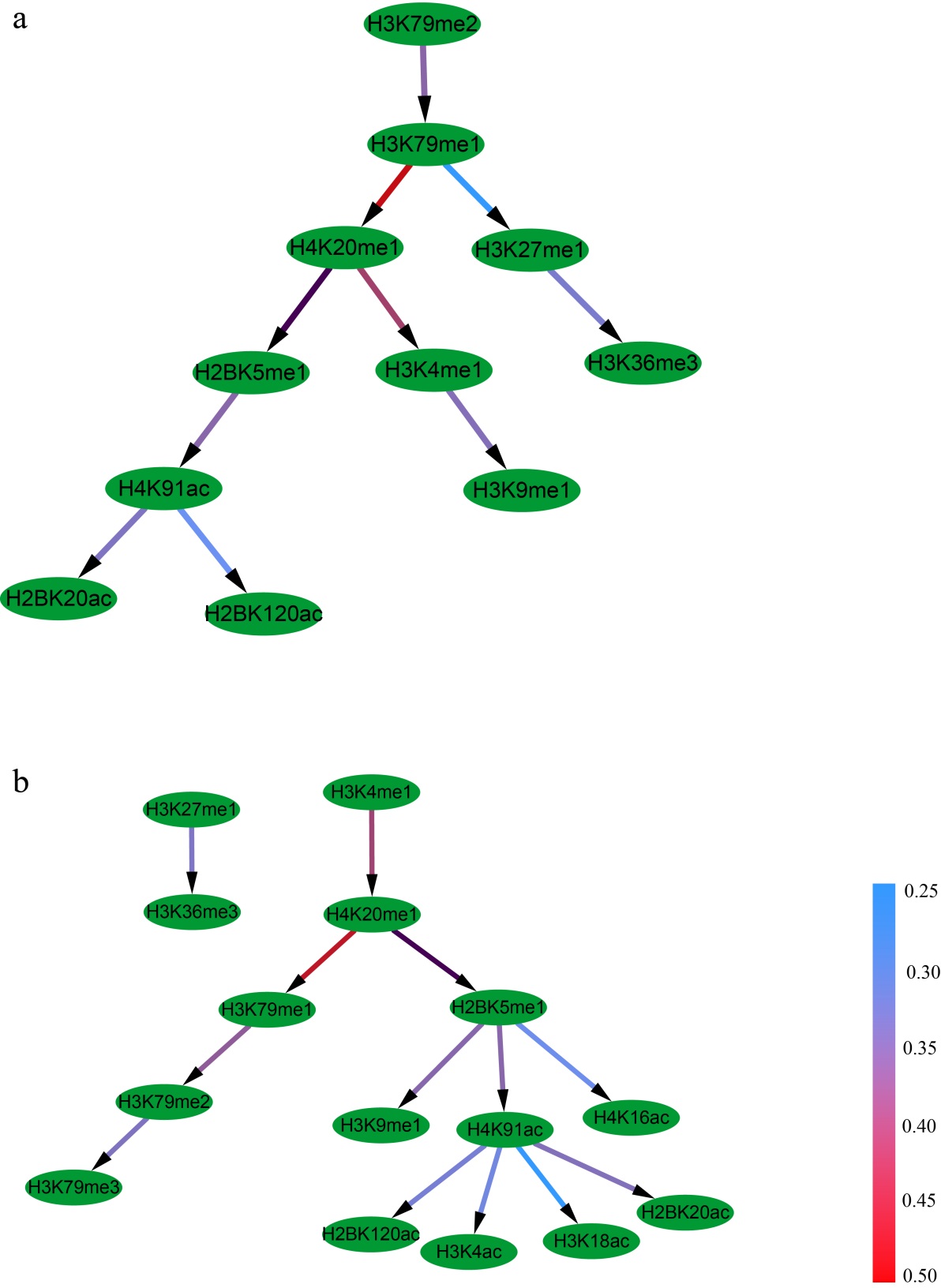


**Figure S6** The Bayesian network of histone modifications in succeeding intronic region of the excluded exon (a) and excluded exon (b).
